# Electrical muscle stimulation in thomboprophylaxis: review and a derived hypothesis about thrombogenesis—the 4th factor

**DOI:** 10.1186/s40064-016-2521-x

**Published:** 2016-06-24

**Authors:** Christos Stefanou

**Affiliations:** ICU, Limassol General Hospital, Eptanisou 2, Agios Nicolaos, 3100 Limassol, Cyprus

**Keywords:** Electrical muscle stimulation, NMES, Thrombosis, DVT, VTE, Nervous system, Endothelium, Vasomotion, Pathophysiology, Thrombogenesis

## Abstract

**Introduction:**

Electrical muscle stimulation (EMS) is an FDA-approved thromboprophylactic method. Thrombus pathogenesis is considered to depend on factors related to components of the vessel wall, the velocity of blood, and blood consistency—collectively known as, the Virchow’s triad.

**Objective:**

The testimony supporting the thromboprophylactic effects of the EMS is reviewed. An emphasis is placed on the fact that, EMS has demonstrated, in certain circumstances, an efficacy rate that cannot be fully explained by the Virchow’s triad; also that, in reviewing relevant evidence and the theorized pathophysiological mechanisms, several findings collectively point to a potentially missed point. Remarkably, venous thromboembolic disease (VTE) is extremely more common in the lower versus the upper extremities even when the blood velocities equalize; EMS had synergistic effects with intermittent compressive devices, despite their presumed identical mechanism of action; sleep is not thrombogenic; non-peroperative EMS is meaningful only if applied ≥5 times daily; neural insult increases VTEs more than the degree expected by the hypomobility-related blood stasis; etc. These phenomena infer the presence of a 4th thrombogenetic factor: neural supply to the veins provides direct antithrombic effects, by inducing periodic vessel diameter changes and/or by neuro-humoral, chemically acting factors. EMS may stimulate or substitute the 4th factor. This evidence-based hypothesis is analyzed.

**Conclusion:**

A novel pathophysiologic mechanism of thrombogenesis is supported; and, based on this, the role of EMS in thromboprophylaxis is expanded. Exploration of this mechanism may provide new targets for intervention.

According to Virchow’s classical triad (Virchow 1856), thrombus pathogenesis depends on factors related to components of the vessel wall (endothelial injury or dysfunction); of the velocity of blood flow (stasis/turbulence); and of the blood consistency (hypercoagulability). Venous thromboembolic disease (VTE), consequential to pathologic thrombus formation, is regarded to result from one or, more frequently, more abnormalities related to these factors; and remains one of the main preventable causes of death (Heit 2015). In-hospital cases account for about three-fourth of the VTE-related fatalities (Cohen et al. 2007).

Electrical muscle stimulation (EMS), the application of electrical current usually through superficial (skin) electrodes placed over the main muscle groups of the lower extremities, inducing repeated muscle contractions, is an FDA-approved method for postoperative VTE prophylaxis. For over half a century, its antithrombic effect has been attributed to acceleration of the venous blood flow of the lower extremities as a consequence of muscle contractions causing compression of the veins; and results in an increase in preload, hence, cardiac output (Doran et al. 1964). This acceleration effect was demonstrated in numerous studies; for instance, in an open-label intra-subject study, peroneal nerve EMS (with pulses of 1 Hz, and a maximum charge of 0.02 mC/pulse) in healthy volunteers caused an increase in peak blood velocities in the peroneal, posterior tibial and gastrocnemius veins by 216, 112 and 137 %, respectively (Griffin et al. 2016).

Hence it is corroborated that, EMS applied on the lower extremities causing brisk muscle contractions during operations remarkably reduces the early postoperative VTE rate. It has been shown that, most VTEs diagnosed during the 1st postoperative week actually occur during the operation time, a fact suggesting that, EMS might always need to be considered during major operations, a time period during which the blood flow is greatly reduced in the lower extremities (Maynard et al. 1991). In accordance, a randomized controlled trial (RCT) that compared EMS (10 Hz, 100 V) plus standard care with standard care alone in 90 patients undergoing total knee arthroplasty, validated this effect, with 11 versus 31 % VTE rate respectively, p = 0.02 (Izumi et al. 2014). In another RCT, peroperative EMS (50 ms, 8 Hz, 40–50 mA) was compared with and found to be superior to dextran 40: the incidence of asymptomatic VTE in the dextran versus the EMS group was, 30 versus 14 % (marginal superiority of the EMS, as 95 % CI 0.90–1.16), and that of silent pulmonary emboli was 35 vs. 10 % (95 % CI 0.11–0.97), respectively (Lindström et al. 1982). Likewise, a third RCT of EMS applied during the operation caused postoperative VTE rate reduction from 10 to 3 % (p < 0.05) (Doran and White 1967); while a fourth RCT in a similar context had two cases of VTE in the EMS versus six in the control branch, reaching no statistical significance (p = 0.28) apparently because it included a highly selected population with low incidence of VTE, and also, the EMS energy delivered had been very low (15–25 V) (Goyal et al. 2012). In the same lines, in a prospective study, calf EMS (30 ms, 15–45 V) during general operation applied to one of the two extremities led to 8 % of VTE occurrence versus 21 % in the opposite (non-stimulated) site, a significant reduction (OR 0.33; 95 % CI 0.15–0.77) (Browse and Negus 1970).

Perhaps the most compelling argument supporting the aforementioned conception of the mechanism of action of the EMS is that, thrombi form almost exclusively in the lower extremity vasculature, a fact about which current literature (own review) does not provide sufficient explanatory evidence of any other difference from that of any other body area further to the slowness of blood flow in them—which the EMS counteracts—, a fact presumably related to gravity, but also to their anatomy, innervation, or even, to a degree, to the relation to the heart (Flinterman et al. 2008; Wright et al. 1951). Indeed, only about 4 % of the VTEs occur in the upper extremities (Muñoz et al. 2008), as noted in a vast postoperative cohort, in which actually most upper extremity VTEs were caused by catheters or local pressure of the veins, mainly from a neoplasm (Flinterman et al. 2008). In parallel to that, the sluggishness of lower extremity blood velocity has been verified, and in the supine horizontal position, that was found to be about half of the one of the upper extremity (Wright et al. 1951).

However, if blood velocity had been the only explanation to all these phenomena, thrombus incidence in the upper would have become equal, or at least somewhat similar, to that of the lower extremities, once blood velocities equalize in all extremities, an occurrence feasibly achieved by lower extremity elevation by 10°–15° while in the supine position, as shown in postoperative patients (Wright and Osborn 1952); or with EMS application, which increases velocities even further—and even more efficiently than the intermittent compressive devices (ICDs, see below) (Broderick et al. 2014). Yet in that position of 15° in the operating room, the ratio of postoperative VTE rate of the lower over that of the upper extremities was, again, very high. In a RCT (with N = 200 patients) in that setting, no thrombi developed postoperatively in the upper extremities versus 12 developed in the elevated lower extremities (a statistically significant difference); versus 7 in the non-elevated lower extremities of a third group of the study that had EMS (0.5 Hz, 120 V) applied on them, during the operation. Hence, neither lower extremity elevation nor EMS application during the operation had been effective enough to reduce the lower extremities’ VTE rate to the (zero, in that study) level of the upper extremities’. Furthermore, despite the fact that the study was not empowered adequately to detect the difference between EMS versus elevation, the statistically insignificant (p = 0.25) difference of 12 versus 7 cases of VTE favoring the EMS group may still be an interesting observation, suggesting that, perhaps, velocity is not the only mechanism of the EMS-induced thromboprophylactic action (Wright and Osborn 1952; Doran et al. 1970). The fact that EMS accelerates blood flow more efficiently than the leg elevation is unlikely to be the explanation of this observation, as will be discussed below (Morris and Woodcock 2004). More importantly, additional RCTs attested that, when mechanical measures are applied on the lower extremities (e.g. during operations), effectively eliminating the pathogenetic factor of blood velocity favoring lower over upper extremity VTE, lower extremity does not merely approximate the upper extremity VTE rate—the latter being extremely low in several postoperative series, especially if no local factors like central lines provoke upper extremity VTE (Browse and Negus 1970; Muñoz et al. 2008; Koo et al. 2014; Mokri et al. 2013).

As noted above, the time during the operation has been blamed for the occurrence of the early postoperative peak of VTEs. A second lower extremity blood velocity nadir has been described by Illingworth et al., which, according to old studies, occurs on (postoperative) day 12, on average. Another paradox appearing here is that, the 2nd peak of pulmonary emboli was observed on day 9, i.e., 3 days earlier to the velocity nadir, under the conditions of those observations (Illingworth and Dick 1949) (notably, postoperative VTE peak in newer series was reported at different timing, but no concurrent blood velocity recordings are available for reference) (Muñoz et al. 2008).

Accumulating evidence supports the appraisal that EMS may also have systemic antithrombic effects, i.e., extending beyond the stimulated extremity. A trend towards statistical significance (p = 0.10 in an underpowered sample for that particular outcome) for VTE reduction in the *contralateral* to the stimulated extremity was noted in a prospective study of intraoperatively applied EMS (50 ms, 0.2 Hz, in which a relative risk reduction of 92 % of the stimulated site was observed, p = 0.0003) (Nicolaides et al. 1972). In some relevance to that, a research team concluded that, electrospasmotherapy (ECT) *that causes contractions* has systemic antithrombic effects, as those were observed only in patients not on muscle relaxants (Worowski et al. 1970). Yet another similar study where muscle relaxants had been administered and the ECT-induced catecholaminergic surge was diminished, circulating plasminogen activator (a factor with antithrombic effect) was found to be elevated after the ECT (Pina and Rodrigues 1974). This elevation is noted after exercise as well (Cohen et al. 1968). It remains unclear if and to what degree there is a true systemic antithrombic effect with EMS, and if that is a consequence of electrical stimulation, the resulting muscle contraction, and or associated catecholamine (Cash and Garder 1972) or blood flow changes (Broderick et al. 2010). Then, an interventional study in chronic spinal cord injury (SCI) patients submitted to lower extremity functional EMS (0.35 ms, 30 Hz, 0–132 mA) found acute raises of antithrombin and cAMP and decreases of thrombin and of ADP-induced platelet aggregation, inducing antithrombic effects; as well as raises of the factors V and X, known to have either anticoagulant or precoagulant role (Sinha et al. 1977; Kahn et al. 2010). In regards to the impact of blood flow per se on anticoagulation, intermittent compressive devices (ICDs) [with an efficacy of a 60 % postoperative VTE reduction in one review that included 15 studies (Urbankova et al. 2006), and equivalent efficacy to peroperative anticoagulant prophylaxis in another review of 14RCTs plus 3 observational studies in which RR was 1.4, 95 % C.I. 0.73–2.64 (Pavon et al. 2016)] which accelerate flow by external vessel compression but cause no muscle contractions, were shown to reduce plasminogen activator inhibitor-1 (Comerota et al. 1997) and to activate fibrinolysis, with no impact on platelets or coagulation factors (Kohro et al. 2005). It remains unproven that EMS is superior to ICDs in VTE prophylaxis, as head-to-head studies for the clinical outcome of VTE are lacking. It is understood that, as EMS stimulates the entire muscle uniformly rather than compressing the vessels from outside, the peripheral to maximal pressure point blood stasis (i.e., in the direction towards the feet) in veins may be avoided with the EMS, as opposed to ICDs. However, those short interruptions of flow were not shown to cause thrombi. Indeed, as discussed below, there is evidence that venous stasis becomes significantly thrombogenic when it lasts for hours, not seconds. Another theory of this EMS [e.g. 0.35 ms, 36 Hz; or 0.07–0.56 ms, 1 Hz, in two studies (Broderick et al. 2014; Jawad et al. 2014)] possible superiority could be that, it accelerates flow more efficiently than ICDs. However, a systematic review of the years 1970–2002 of all mechanical non electrical thromboprophylactic measures reported that, peak velocity does not affect efficacy (Morris and Woodcock 2004). Despite the fact that EMS had not been included in the review, it can be extrapolated that, relying on hemodynamic parameter modifications induced by mechanical measures and coming to conclusions about their relative efficacy is not appropriate. Finally, combining ICDs with EMS led to synergic VTE prophylactic effect, a fact that would not be expected should both methods acted by the same mechanism and only, i.e., by flow acceleration (Kopetzky 1994). Concluding this paragraph, some evidence suggests that EMS may have systemic antithrombic effect; may theoretically be superior to ICDs; and its mechanism of action may not solely be related to hemodynamic alterations.

A study in neurosurgical patients in which peroperative EMS (50 ms, 8 Hz, 40–50 mA) was followed by postoperative dextran infusions every 48 h, this (dextran + EMS study group) approach led to more VTE reductions when the spinal cord rather than the brain had been the diseased site of the nervous system (Boström et al. 1986). Further to this point, it has been observed that, injury of the spine in major trauma patients acutely raises VTE rate by 20 %; yet when the spinal cord is also injured, representing a risk factor of 8.6, the likelihood of VTE development was >80 %, in a large trauma cohort of average incidence of VTE of 58 % (Geerts et al. 1994). Deceptively, VTE incidence may be >50 % in SCI patients, with reports varying between 19 and 100 % (Teasell et al. 2009), being much higher than that expected from flow deceleration alone. Let here point out that, sleep as a cause of hypomobility, yet not disturbing peripheral nerve function, is not a known cause of VTE (Heit 2015). Overall, it appears that EMS has more to offer in conditions of complete absence of neural supply to the lower extremities; and that the latter, when occurs, greatly increases the VTE rate (Boström et al. 1986; Nicolaides et al. 2013).

EMS (3 ms, 1.75 Hz, 0–120 V) applied after multiple trauma twice daily was ineffective in VTE prevention (Velmahos et al. 2005); but when applied at least five times daily (Lobastov et al. 2013) (in 20 min sessions, 0.03–2 Hz, in high risk patients, with the FDA-approved device *Veinoplus* (Griffin et al. 2010), in addition to standard prophylaxis), VTE rate was reduced from 25 to 2.5 % (p = 0.007) (Lobastov et al. 2014). In the same lines, an RCT in severe acute SCI patients found a very remarkable decrease of VTE incidence by adding lower extremity EMS (0.05 ms, 10 Hz) applied 23 h daily to prophylactic dose of unfractionated heparin (UH) (1 in 15 patients in the EMS + UH vs. 8 in 16 patients in the UH group, p < 0.05), applied <2 week after injury for 4 weeks (Merli et al. 1988).

Vasomotor phenomena were observed or increased by the EMS (0.2 ms, 1–5 Hz, 1–40 mA) beyond the degree expected by mechanical compression and metabolism augmentation alone (Tucker et al. 2010). At this juncture let us also recall an important FDA-approved EMS indication, i.e., in pressure ulcer prevention, where it appears to interfere with local tissue reactions, such as, increasing local oxygenation, perfusion, and or to exert anti-infectious effects (Polak et al. 2014). The EMS potentiates galvanic sensing of local healing cells and their reaction to transepithelial potential elimination of the wounded epithelium (Kawasaki et al. 2014). The effects of the EMS (0.07–0.56 ms, 1 Hz, 27 mA) on the microcirculation of the stimulated extremity, to a degree more pronounced than that caused by ICD, were also demonstrated in a RCT that utilized laser Doppler fluximetry (Williams et al. 2014). Another study of the acute effects of EMS in ICU patients demonstrated an increase of endothelial reactivity and a decrease of the vascular reserve, as evaluated by near infrared spectroscopy (NIRS), signifying local and systemic effects on skeletal muscle microcirculation (Angelopoulos et al. 2013). Vasomotor systemic phenomena were shown in other studies as well (Jawad 2012).

## In summary

EMS, like any mechanical measure, accelerates blood flow and this appears to mediate its thromboprophylactic effect, which has been demonstrated, particularly when applied peroperatively, in several studies. Indeed, the high VTE rate in the lower extremities in comparison to other body areas appears to relate to their slow venous circulation, which the EMS counteracts. However, as reflected on evidence (and occasionally on critiques, since the 40s) (Doran et al. 1964; Illingworth and Dick 1949) this as a standalone explanation does not suffice to illuminate a considerable percentage of findings of related studies to date. For instance, when all extremities’ venous blood flow becomes equal, VTE rates remain quite dissimilar between the upper and lower extremities (Doran et al. 1970); and when EMS had been used to make venous blood velocities between extremities equal, the VTE reduction in the lower extremities was likely more pronounced than the reduction that had been achieved by a different method (underpowered study, statistical trend reached, and clinically notable). Then, despite velocity manipulations, never has the VTE rate of the lower extremities approached the approximately 22-fold lesser (Muñoz et al. 2008) levels of that of the upper (Howie et al. 2005). Postoperative pulmonary emboli incidence timing pattern did not match that of blood velocity. EMS prophylactic efficacy may extend to the non-stimulated extremity (Nicolaides et al. 1972; Broderick et al. 2010); furthermore, EMS appears to exert systemic effects. EMS may be superior to ICDs in VTE prophylaxis, yet clinical evidence is limited; but the two methods applied together had synergistic antithrombic effects, making the perception that their mechanism of action is identical poorly acceptable (Kopetzky 1994). In acute injury/insult of neural supply to the lower extremities, VTE rate increases dramatically beyond that expected from flow deceleration alone—such as in cases of paralysis not involving the nerves, or during physiologic sleep. EMS has a lasting post-sessional effect that makes it a meaningful intervention if applied ≥5 times daily (Velmahos et al. 2005; Lobastov et al. 2013; Griffin et al. 2010) or continuously (Merli et al. 1988) [with the remarkable exception of EMS during the operation (Izumi et al. 2014; Lindström et al. 1982; Doran and White 1967; Goyal et al. 2012; Browse and Negus 1970), as most early VTEs occur during that period (Maynard et al. 1991)]. EMS may interact with perivascular or tissue factors or vasomotor processes (Tucker et al. 2010; Polak et al. 2014; Kawasaki et al. 2014). Overall, its mechanism of action does not seem to be limited to hemodynamic modifications. These facts raise the possibility that neural factor is of immense importance in thrombus pathogenesis (Schneck 2014). All pieces put together may lead to a possible conclusion, a hypothesis presented below.

## A hypothesis

### Neural supply to the veins provides direct antithrombic effects

This would constitute a 4th factor in thrombus pathogenesis (i.e., one beyond Virchow’s triad). Periodic neural activity may cause variations in venous diameter AND/OR influence the veins by transmitters that induce local interactions, resulting in antithrombic mechanical and/or chemical (neuro-humoral) effects. Presence of a neurogenic pacemaker in vasomotion is likely. The neural cells involved may recite in or relate to the sympathetic neural system (SNS) or function, or other non-noradrenergic (see discussion section) neurons, which interact with vascular wall components. Furthermore, muscle contractility can relate to corelease of endocrinologically and immunologically (myokines) active factors (Raschke et al. 2013; Pedersen and Febbraio 2008). A biologic role in thomboprophylaxis of the latter is possible as well. Any or combination of these mechanisms may be involved in the functions of the 4th factor.

A direct or indirect insult to this process or a decline of its periodic output (which can even be an adaptation to the patient’s condition) is expected to increase thrombogenicity; also to cause blood stasis, that further worsens the precoagulant state. This concept differs from the current view of the vascular wall as a static structure in thrombogenesis; but rather, wall motion, perpendicular to the blood flow, mechanically fragments a *primary* thrombus that begins to form; and/or vascular wall factors being released in response to neurotransmitters, by diffusion reach an adjacent developing thrombus causing chemical destabilization.

#### EMS may stimulate or substitute the 4th factor

 This could be an efferent effect through neurons transferring the pulses to the periphery, or have an initial afferent path to the CNS, followed by reflex responses. Let cite, EMS is shown to have *central effects*, which cause central neural adaptations. EMS pulses diffuse within the CNS; and part of the stimulation returns to the periphery, as described/evidenced in Hortobagyi and Maffiuletti model (Hortobagyi and Maffiuletti 2011).

As its mechanism of action is not limited to hemodynamic modifications, EMS may be combined with other mechanical measures with synergistic effect (Kopetzky 1994).

A primary thrombus requires several hours to stabilize (chemically and mechanically) (Schulz et al. 2013). This might be the explanation of the fact that, EMS applied less frequently than 5 times daily is clinically ineffective (with time gaps sufficient for thrombus formation). Intraoperatively applied EMS is an exception, as most VTEs occur during surgery; hence, one single session may suffice to substantially reduce the early VTEs. Some percentage of prevention of thrombus formation and or related thrombolytic cascade activation might explain the coagulation factor changes induced by the EMS in the aforementioned studies. Another explanation to those findings could be a direct effect from the EMS, which, as a substitute to the 4th factor, might have induced vessel wall release of factors contributing to systemic antithrombic effects. A third possibility could be through the release of myokines.

The likelihood that the processes of the 4th factor are not affected during sleep could explain why sleep, contrary to general anesthesia or nerve injury-related immobility, is not thrombogenic.

**Fig. 1:**
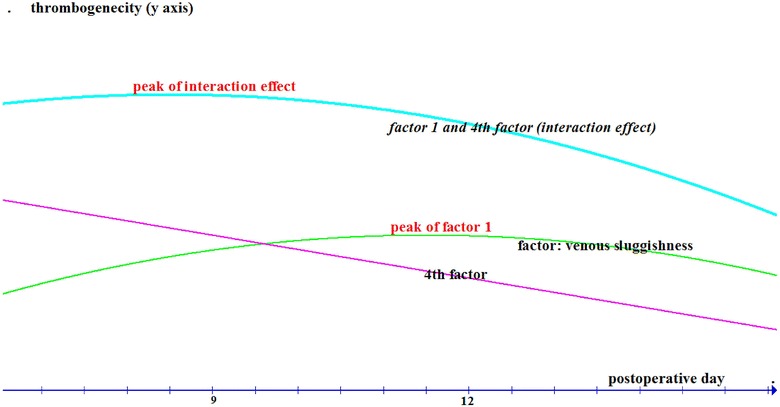
Factors contributing to thrombogenecity after general surgery [thrombogenecity vs. time (days)]

During the 2nd postoperative week, two contributing thrombogenic processes could have evolved under the conditions described by Illingworth et al., i.e., a progressive decline of the thrombogenicity due to the recovery of the 4th factor and a “reversed-U-shaped” (over time, i.e., peak) thrombogenic process due to the slow recovery of the (U-shaped, i.e., nadir) blood velocity, ended up in a cumulative thrombogenicity peak on day 9 rather than day 12 (assuming that the inflammation induced from surgery does not contribute remarkably to thrombogenesis during that period—at least to a degree different between day 9 and day 12) (Fig. 1).

This (presently described) mechanism is not the only one through which EMS exerts VTE thomboprophylaxis. Like exercise, it accelerates venous flow (by vessel compression, heart rate acceleration, increase of preload, of metabolism etc.), it may cause systemic release of anticoagulants or and anti-inflammatory mediators etc. (pleotropic effects of EMS/exercise) (Raschke et al. 2013; Petersen and Pedersen 2005).

It is possible that there is a difference between *innervation*—which correlates to the 4th factor component—of the vessels of the upper extremities versus the lower extremities. Experiments in mice have demonstrated that, gravity may be causing long-standing neural adaptations, functional and anatomical, at the nerves of the vessels of the extremities; and these will differ in the upper from the lower extremities (Monos et al. 2001). This would partly explain the difference in VTE rate among the two, which cannot be explained solely with hemodynamic terms; but the addition of the 4th (neurogenic) factor may provide an answer.

As with any hypothesis, it requires further research. The anatomical representation and detailed functionality of the 4th factor need to be explored. Importantly, these need to be looked for in a system characterized by periodic activity, such as, the sympathetic neural system and its interconnections with the vascular neural plexus (see “Discussion” below).

## Discussion

Some basic pathophysiology in regards to the interactions involving perivascular neurons, vasomotion and the endothelium in hemostasis is cited, and may need to be considered in hypothesizing the potential functionality of the 4th factor.

Communication between neurons of the vascular wall neural plexus is mediated by adrenergic, cholinergic, purinergic (e.g. ATP), peptidergic, and nitrergic (of nitric oxide, NO) neurotransmitters (Westcott and Segal 2013). The perivascular nerves, after calcium influx in them, release transmitters that by diffusion or heterocellular communication—mainly via gap junctions—reach adjacent neural, endothelial and smooth muscle cells (SMC). Efferent vasoconstrictive sympathetic fibers, tonically activated, predominate in the plexus. Noradrenaline, the main neurotransmitter, causes contraction of the SMC via the inositol triphosphate/calcium pathway; while the same molecules may diffuse to endothelial cells, causing reactions like NO release, which then diffuses to the SMC moderating their preceded contraction (Kansui et al. 2008). During sympathetic neural system (SNS) stimulation, other molecules may be coreleased, such as, ATP (acting on the endothelium) (Burnstock 2014) and neuropeptide Y (Hirsch and Zukowska 2012). The non-noradrenergic systems appear to have weak, modulatory role. Sensory fibers, also capable for efferent transmission after noxious or mechanical stimuli, generally induce vasodilatation, via substance P, calcitonin-gene-related-peptide-alpha, which acts via the G-protein/cAMP pathway, and other neurotransmitters (Donoso et al. 2012).

Vasomotion, best studied in arteries, is the periodic spontaneous change in vascular diameter, thus regulating local tissue perfusion, blood pressure and other essential functions. It is synchronous among remote SMC, causing the entire vascular wall to oscillate. Evidence supports that it initiates with intermittent asynchronous sarcoplasmic reticulum release of calcium, thus activating a cell membrane potential/ion current by involving either inositol triphosphate or ryanodine receptors. Vasomotion occurs when the two current events synchronize. Adjacent cell coupling via gap junctions or indirect coupling appears essential in achieving synchrony of intracellular oscillations of calcium influx. The authors of the theory suggest that, the pacemaker of the vascular wall can be envisaged as a diffuse array of individual cytosolic oscillators that become entrained by a reciprocal interaction with the cell membrane (Peng et al. 2001; Koenigsberger et al. 2004). A (cGMP-dependent calcium-activated) chloride current may be involved in synchrony between SMC in some vessels (Matchkov 2010), while in others, with high level of coupling between SMC, endothelial, and SMC with endothelial cells, voltage-dependent calcium channels are also involved, and generation of a depolarizing current occurs (Nilsson and Aalkjaer 2003). Alternatively, a vasoconstrictor-like noradrenaline—may induce tonic synchronous depolarization. The role of the endothelium in synchronization may depend on whether myoendothelial gap junctions are abundant; if so, it coordinates the electrical responses between neighboring SMC; if not, it interacts through the release of vasodilators affecting the SMC (Haddock and Hill 2005). In comparison to the arteries, most veins contain more adrenergic terminals, distributed in the media (Birch et al. 2008) and their responses to sympathetic stimulation is brisker (Racchi et al. 1997) causing reduction in their capacitance (Fallick et al. 2011).

Endothelium in hemostasis under non-traumatic conditions releases NO, prostacyclin and adenosine, which cause vasodilation, and cAMP and cGMP release from the platelets, which inhibit their activation. The venous wall releases plasminogen activator in response to trauma or blood stasis. Exogenous and endogenous coagulation pathways are depended on the direct contact of coagulation factors with the endothelial cells (Pettigrew 2001; Geenen et al. 2012).

Hence thrombogenesis is a composite of mechanical and chemical interactions involving neurons, the endothelium and components of vasomotion. Τhe 4th factor could be implicated in more than one processes required for thrombus formation and stabilization.

In terms to stimulation parameter to be used for VTE prophylaxis, the pool of evidence does not demonstrate any particular pattern providing superiority. After extensive literature review, no comparisons between different current characteristics were found in terms of VTE prophylactic efficacy. Extrapolating parameter-related conclusions from other applications of EMS (such as, for ICU-acquired weakness) does not appear appropriate, since there is no suggested identical mechanism of action with VTE prophylaxis. Furthermore, the fact that quite different parameters appeared effective across different settings and studies (Table 1), as well as the fact that hemodynamic parameters correlate poorly with VTE prophylactic effects (Morris and Woodcock 2004) may indicate that, what mostly matters is the duration of the application over the 24 h period. Thus, pending further evidence, it appears reasonable to select a current intensity (dose) causing brisk contractions, if possible, but certainly be comfortable enough to allow application for most hours per day, during the entire period in which the patient is at moderate or severe risk of VTE. Any suggestion of other parameters (frequency, pulse shape, pulse duration, on: off time, ramp up/down etc.) has not been investigated sufficiently, hence, any suggestion is much likely to be proven false.

**Table 1:** Parameters of EMS aiming to provide VTE prophylaxis, used across different studies, where reported

*Parameter*												
References	Griffin et al. (2016)	Izumi et al. (2014)	Lindström et al. (1982)	Goyal et al. (2012)	Browse and Negus (1970)	Doran et al. (1970)	Nicolaides et al. (1972)	Broderick et al. (2014)	Jawad et al. (2014)	Boström et al. (1986)	Velmahos et al. (2005)	Merli et al. (1988)
Dose	0.02 mC^+^	100 V	N	15–25 V	15–45 V	120 V	N	N	27 mA	~45 mA	≤120 V	N
Frequency (Hz)	1	10	8	N	N	0.5	0.2	36	1	8	1.75	10
Tx period	N	OR	OR	OR	OR	OR	OR	N	N	OR	PT	PT
Pulses (ms)	N	0.5	50	N	30	N	50	0.35	~0.06	50	3	0.05

N, not stated/not relevant; +, maximum charge/pulse; OR, peroperatively; Tx, treatment; PT, post trauma period

### Further vascular benefits; and hurdles related to the EMS

The present review focuses on the beneficial effects of the EMS on the veins. Further to that, there is strong evidence that the beneficial effects of the application are more global, involving all vessels via additional mechanisms, likely most importantly those inducing mobilization of endothelial progenitor cells from the bone marrow (Stefanou et al. 2016). The latter are considered to exert healing effects on the injured endothelium by the insults of illness. This regeneration potential may be of vital clinical importance (Balistreri et al. 2015).

Probably the most bothersome and discussed problem related to the EMS is tolerance—namely fatigue and pain. A systematic review on its use for thomboprophylaxis, which included 21 studies, concluded that, in reference to modern devices, EMS is *generally associated with an acceptable tolerability, potentially leading to good patient compliance.* Newer EMS methods do not induce painful contractions as the case had been with the devices used intraoperatively during the 80s and 90s (requiring general anesthesia); and may be applied multiple times daily; yet this requires further evaluation (Hajibandeh et al. 2015).

In the context of application of bundles of measures with pleotropic effects in the ICU, step-down unit and rehabilitation, EMS arises as a potent advantageous modality in multiple levels. It has shown to exert beneficial metabolic effects (Hamzaid and Davis 2009); and likely contributes to the prophylaxis from ICU-acquired weakness (Gorgey et al. 2012) and associated earlier weaning from the ventilator (Routsi et al. 2010); the healing of deep tissue injury (Franek et al. 2012); the post-injury nerve regeneration (Xu et al. 2014); it improves endothelial function (as demonstrated by the flow-depended brachial artery dilatation); and exerts anti-inflammatory effects (as demonstrated by TNF-alpha, s-ICAM-1, s-VCAM-1 and TNF-α/IL-10 ratio reduction) (Karavidas et al. 2006).

## Conclusion

Evidence supports the hypothesis that, neural supply to the veins provides direct antithrombic effects, a factor not included in Virchow’s triad. EMS appears to be one mechanism that, via neurogenic pathways, influences this 4th factor, thus suppressing thrombogenesis. As VTE remains one of the most preventable causes of in-hospital mortality, this hypothesis and factor as well as ways to affect it need to be explored. In addition, EMS needs to be investigated with well-designed RCTs for its possible *additive* effect on each thromboprophylactic measure, including the mechanical ones, as its implementation may save numerous lives.

### Implication

By ‘replacing’ the lost periodic neural supply to the veins, or by enhancing so, EMS acts not only by blood velocity modifications (as part of the Virchow’s triad) but also by the 4th factor. Although EMS might have a secondary role in thomboprophylaxis, the prevalence and mortality of VTE are so high that it may save a great number of lives; and since it may target a unique aspect of the disease pathogenesis, its role perhaps will not be substituted by alternative mechanical measures; but it may be applied in combination with the latter, providing additive effects. Further to that, the most important implication of identifying and exploring the related pathophysiology of *the 4th factor* as part of thrombus pathogenesis is that, after scrutiny to fully characterize, quantify, and modify, with research focused on specific questions, new thromboprophylactic strategies (in addition to EMS) may be found based on this factor; and applied to patients under conditions in which the 4th factor is affected or when the risk of VTE is high, such as, after major operations, trauma, or in ICU patients.
